# Evaluation of Antioxidant Activity and Physicochemical Characterization of Walnut (*Juglans regia* L.) Oil

**DOI:** 10.3390/ijms27104390

**Published:** 2026-05-14

**Authors:** Marilena Viorica Hovaneț, Mihaela Afrodita Dan, Denisa Margină, Anca Ungurianu, Adina Magdalena Musuc, Emma Adriana Ozon, Cornelia Bejenaru, Adriana Rusu, Mihai Anastasescu, Veronica Bratan, Claudia Maria Guțu, Daniela Luiza Baconi, Dumitru Lupuliasa, Gabi Topor

**Affiliations:** 1 Faculty of Pharmacy, “Carol Davila” University of Medicine and Pharmacy, 6 Traian Vuia Street, 020945 Bucharest, Romania; marilena.hovanet@umfcd.ro (M.V.H.); mihaela.dan@umfcd.ro (M.A.D.); denisa.margina@umfcd.ro (D.M.); anca.ungurianu@umfcd.ro (A.U.); emma.budura@umfcd.ro (E.A.O.); claudia.gutu@umfcd.ro (C.M.G.); daniela.baconi@umfcd.ro (D.L.B.); dumitru.lupuliasa@umfcd.ro (D.L.); 2 Institute of Physical Chemistry–Ilie Murgulescu, Romanian Academy, 060021 Bucharest, Romania; arusu@icf.ro (A.R.); manastasescu@icf.ro (M.A.); vbratan@icf.ro (V.B.); 3 Faculty of Pharmacy, University of Medicine and Pharmacy of Craiova, 2 Petru Rareş Street, 200349 Craiova, Romania; cornelia.bejenaru@umfcv.ro; 4 Department of Pharmaceutical Sciences, Faculty of Medicine and Pharmacy, “Dunarea de Jos” University of Galati, 35 Al. I Cuza Street, 800010 Galati, Romania; gabi.topor@ugal.ro

**Keywords:** *Juglans regia* L., sunscreen protection factor, GC-MS analysis, vegetable oils, natural products

## Abstract

*(1)* The growing interest in the use of natural and sustainable ingredients highlights the investigation of vegetable oils in dermato-cosmetic applications. In this context, the vegetable oil obtained from walnut (*Juglans regia* L.) is of actual interest due to its composition rich in unsaturated fatty acids. The aim of the present study was to investigate and characterize walnut oil from a physicochemical, structural, and rheological point of view. *(2)* The oil was obtained by a cold pressing process from walnut seeds, with a yield of about 51.03 ± 1.41%, and subsequently analyzed by complementary methods. *(3)* The results show an acceptable physicochemical profile, characterized by appropriate values of density, pH, and spreadability. The oxidative stability indicated a moderate resistance to degradation, specific to oils rich in polyunsaturated fatty acids. Fourier infrared transform spectrometry (FTIR) analysis confirmed the presence of functional groups characteristic of triglycerides, without indications of advanced oxidation, and atomic force microscopy (AFM) investigations revealed a heterogeneous morphology. The rheological properties indicated a pseudoplastic behavior, favorable for topical application. The determination of heavy metals confirmed the safety of the raw material for the intended dermato-cosmetic use. While arsenic levels were slightly above the strict Codex Alimentarius limits for foodstuffs, all values remained within the safety ranges established for cosmetic ingredients. A total of six fatty acids were found in cold-pressed walnut oil, determined using GC-MS methods. The number of compounds identified in the silylated sample was found to be 17. The antioxidant activity determined using DPPH and ABTS methods was generally considered good and relatively stable over time. The measured sun protection value (SPF) demonstrates a favorable capacity to act as a photoprotective ingredient against ultraviolet (UV) radiation. *(4)* Overall, the results demonstrate that walnut oil presents adequate physicochemical and structural properties, supporting its further use as a potential cosmetic raw material.

## 1. Introduction

The most commonly used active ingredients in the skincare and cosmetic industries are waxes, esters, mineral oils, natural butters, and fats. These substances are commonly used (i) as emollients to moisturize and act as a skin barrier against moisture loss or to create a natural protective layer, to spread quickly, (ii) as nourishing agents to provide long-term hydration, (iii) as solubilizers to reduce surface tension and dissolve insoluble ingredients, (iv) as agents for controlling the viscosity and stability of formulations, or (v) as pigment-dispersing compounds. In recent years, the need to develop cosmetic products primarily based on green, natural, and sustainable ingredients, especially those of plant origin, has been growing. Research has focused on identifying new bioactive compounds that meet both consumer demand for natural products and industry requirements for sustainability, while having minimal ecological impact [1,2]. In this context, the sustainable use of natural resources, as renewable and eco-friendly compounds, represents an important step in current dermato-cosmetic research. Vegetable oils have been used since ancient times in beauty and skin care products, due to their versatility and beneficial properties. Ancient Egyptians were the first to use the oil extracted from flax seeds or radishes for therapeutic, aesthetic, or nutritional purposes [3,4]. They are obtained from natural sources such as flowers, seeds, fruits, and nuts (e.g., olive oil, sunflower oil, sesame oil, grape seed oil, coconut oil, and peanut oil). The oils extracted from plant origin contain mostly 95–98% triglyceride (saturated fatty acids, monounsaturated fatty acids (MUFAs), polyunsaturated fatty acids (PUFAs)) and 1% mono and diglycerides, namely glycerolipids and non-glycerolipids composed by tocopherols, vitamins, water, free fatty acids, and phenolic compounds [5,6].

The oxidation stability of vegetable oils is an important factor that is influenced by the composition of fatty acids. In particular, edible vegetable oils exhibit faster oxidation when their composition contains a higher quantity of polyunsaturated fatty acids compared with saturated ones [7,8]. The vegetable oils gained attention in utilization as active ingredients in sunscreen formulations used for skin protection against ultraviolet (UV) radiation. UV radiation causes the most skin cancer in the world by distorting the DNA of human skin cells [9]. Many vegetable oils extracted from plant seeds (e.g., shea nut, green tea, jojoba oil, pomegranate seed, and raspberry seed) contain natural compounds with potential UV-absorbing and photoprotective properties, which make them possible candidates for use in the cosmetic industry [10].

Walnut (*Juglans regia* L.), the oldest tree, has been known since 7000 BCE in Persia and belongs to the *Dioscaryon* section [11]. The walnut seeds are widely consumed due to their lower cholesterol content. The fat content of walnut oil is between 52 and 74 percent of lipids is about 70%, and the literature data reported compounds like 15.2% proteins, polyunsaturated fats (linoleic acid, C18:2 (ω-6), and α-linolenic acid, C18:3 (ω-3)) [12], monounsaturated fats (oleic acid, C18:1), 6.7% dietary fiber, 13.7% carbohydrates, and 1.8% ash content [13,14,15]. The presence of diversity and higher content in bioactive compounds, such as polyphenols and tocopherols, makes walnut oil a promising candidate for widespread use in medicine, agriculture, and food domains. Due to its recognizable anti-inflammatory [16] and antioxidant capacity, walnut oils have been extensively used for the treatment of intestinal, digestive, and ulcerative colitis and inflammatory bowel diseases [17,18,19,20]. Also, due to its improved antioxidant capacity, walnut oil has been extensively used in cosmetics as an antiaging compound [21] and skin emollient [22].

Different extraction methods are reported in the literature for obtaining walnut oils, including solvent extraction (methanol and chloroform), cold pressing, maceration in solvent, Soxhlet extraction, and supercritical carbon dioxide (SC-CO_2_) extraction [11].

The selection of the most appropriate extraction method is based on the required properties of the obtained oils and the impact of the extraction process on the quality and amount of the important components and lipids from the processed oil. It must avoid the degradation of antioxidant compounds, yielding a pure oil with fewer impurities, and improved extraction efficiency [23]. Soxhlet extraction leads to lower linoleic acid in oil and also a lower content of tocopherol. Also, the method leads to a chemical contamination of the oil [24]. SC-CO_2_ extraction has several disadvantages, namely, decreasing the stability of oil, losing volatile compounds, and obtaining no good yield. The cold pressing method is the most commonly used method widely used due to its safety (no chemicals and heat treatment), a high retention of flavor and color, without minimal degradation of functional compounds and higher nutritive properties [25].

In recent years, walnut oil has attracted increasing attention due to its high content of unsaturated fatty acids and bioactive compounds, with reported nutritional and cosmetic benefits. However, most previous studies have focused mainly on nutritional properties or partial compositional characterization, while limited information is available regarding region-specific cold-pressed walnut oils intended for dermato-cosmetic applications. In particular, integrated studies combining chemical profiling, antioxidant activity, oxidative stability, and photoprotective potential have not been extensively studied. In this context, the novelty of the present study lies in the comprehensive evaluation of cold-pressed walnut oil obtained from walnuts cultivated in a specific region of Romania. Furthermore, this study combines GC-MS characterization, antioxidant activity assessment, oxidative stability index determination, and in vitro sun protection factor (SPF) evaluation to explore the property as a dermato-cosmetic raw material through a multi-analytical approach. To the best of our knowledge, such an integrated investigation has been insufficiently addressed in the existing literature. This study was designed as a representative case study to evaluate the potential of a region-specific walnut oil as a cosmetic raw material.

## 2. Results and Discussion

### 2.1. Physical Constants of Juglans regia L. Oil (JRO)

From 100 g of vegetal products, a 51.03 ± 0.89 g yield of lipid-rich oil from *Juglans regia* L. was obtained. No further refining process was made on the obtained oil. The extraction yield was 51.00 ± 1.41%. The cold pressing process yield of 51% is consistent with the oil extracting process reported by Gao et al. [26] and Masoodi et al. [13]. The obtained oil was slightly colored, with a pleasant smell and a neutral reaction. The yield is appropriate, and the oil’s organoleptic characteristics support its use in phytotherapy. The physicochemical properties of JRO were determined in accordance with the European Pharmacopeia [27], and the results are listed in Table 1:

The value of the refractive index is about 1.4719, which demonstrates the high degree of unsaturation characteristic of plant seed fatty oils. The value is in accordance with other unsaturated fatty oils studied in the literature [13]. Arafat et al. [28] determined a value of refractive index of 1.4556 for palm oil and 1.4702 for olive oil. This value also reflects the purity of the oil and shows that the analyzed JRO sample has good physical characteristics. Also, Masoodi et al. reported a value of refractive index for a walnut oil extracted using the cold press method to be 1.437 ± 0.02. [13] The density value of 0.929 ± 0.030 g/m^3^ is justified for vegetable oils with a composition of higher unsaturated fatty acids [29]. The viscosity of 9.22 ± 0.01 ^o^Engler is specific to the flow behavior of JRO. The pH value is 5.00 ± 0.02 and is in the typical 4–6 range, having compatibility with the pH skin and characteristic for promoting skin health [30,31].

The acid value, which represents the amount of KOH (mg KOH) required for neutralizing the free fatty acid fraction in 1 g of oil, signifies a method to determine the content of free fatty acids. The acid value of JRO of 7 mg/g characterizes the free fatty acids that are present in the oil. This value is comparable with the value for African oil bean reported by Odoemelam et al., which was determined to be 7.01 mg/g [32].

The iodine index is an indication of the degree of unsaturated fatty acids. The iodine value determined for JRO is 76.90 ± 0.02 (I_2_ g/100 g oil), which indicates a medium degree of unsaturation.

The saponification (esterification) index (mg KOH/1 g oil) is an indication of the presence of fatty acids with lower molar mass, which give a higher saponification index, and fatty acids with higher molar mass give a lower saponification index [33].

### 2.2. GC-MS Analysis

**Figure 1:**
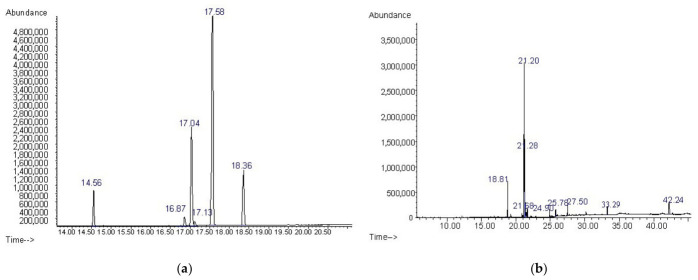
(**a**) GS-MS analysis of fatty acid contents of JRO and (**b**) of compounds from silylated samples.

Table 2 and Table 3 (and Figure 1a,b) represent the total number of fatty acids of JRO and the total number of compounds from the silylated oil sample detected by GC-MS.

A total of six fatty acids were identified in JRO, determined by GC-MS, such as palmitic acid (C16:0, 6.394%), stearic acid (C18:0, 1.905%), oleic acid (C18:1, 20.019%), linoleic acid (C18:2, 58.582%), and α-linolenic acid (C18:3, 12.253%). The linoleic and α-linolenic acids together represent more than 70% of the total fatty acid content. This proves that the walnut oil is a representative source of polyunsaturated fatty acids. The results are in agreement with those obtained by Gao et al. [26]. Gao et al. reported a composition of the main five fatty acids of about 66.56 ± 0.02% for C16:0, 3.24 ± 0.01% for C18:0, 14.64 ± 0.02% for C18:1, 67.05 ± 0.03% for C18:2, and 8.31 ± 0.02% for C18:3 obtained for a walnut variety from Xiangling by cold pressing. As saturated fatty acid palmitic acid (C16:0) is present in 6.394%.

The results of GC-MS analysis of silylated JRO sample reveal a mixture of free unsaturated fatty acids and sterols. The higher polyunsaturated acids presented are free linoleic and oleic acid (41.679% and 22.630%, respectively) and silylated sterols. From Table 3, it can be observed that the two significant phytosterols, the important bioactive compounds in walnut oil, β-sitosterol and stigmasterol, were present in the composition of walnut oil (β-sitosterol of 8.588% and stigmasterol of 1.101%), accounting for over 10% of the total compounds identified for the silylated JRO sample. Findings indicate that β-sitosterol extracted from walnut oil has an inhibitory effect on MCF-7 cell proliferation [34]. The aldehydes and unsaturated hydrocarbons are present in lower concentration (<0.5%).

The results for α-linolenic acid dosage are presented in Table 4.

The relatively low content of α-linolenic acid compared to linoleic acid is typical for walnut oil, where omega-6 fatty acids predominate. However, its presence in 2.5 mg/100 g concentration confirms the functional character of the oil, contributing to the omega-6/omega-3 balance, an essential parameter in assessing the nutritional quality of the walnut oil [35,36].

### 2.3. Heavy Metals Content

The results are expressed in both measurement units (µg/L and µg/g) and are illustrated in Table 5.

The arsenic concentration detected (0.145 mg/kg) is slightly above the maximum limit of 0.10 mg/kg established by Codex Alimentarius for edible oils [37]. However, this study focuses on the topical application of walnut oil. In the cosmetic industry, safety limits for heavy metals in raw materials are generally higher than for food (often up to 2 mg/kg for arsenic in various international guidelines), as skin absorption of inorganic arsenic is significantly lower than gastrointestinal absorption. Therefore, based on the intended dermato-cosmetic use, the oil presents a favorable safety profile with no significant risk of systemic toxicity. The results obtained are consistent with the values reported in the recent literature, whereas concentrations in edible sesame oil are identified at a concentration of 15.18 µg/kg [38].

JRO sample content of lead falls in approximately half of the maximum permitted limit of 100 µg/kg (0.1 mg/kg) established by FAO/WHO [39] and CODEX [40] for heavy metals in vegetable oils and fats. Compared to the established limits, the analyzed sample falls well below the maximum permissible threshold, demonstrating compliance with the safety requirements imposed at the European level. The value obtained is also consistent with data reported in the recent literature, where typical Pb concentrations in vegetable oils [41,42,43]. In corn oil from Iran, Pb^2+^ was identified in a concentration of 0.099 mg/kg [44]. This confirms the accuracy of the method used and the absence of external contamination in the analyzed samples. Therefore, the HNO_3_/H_2_O_2_ mineralization method in the Ethos Easy microwave system, combined with GFAAS detection, proved to be efficient, reproducible, and sensitive for the determination of heavy metal traces in vegetable oils. In conclusion, the analyzed oil can be considered uncontaminated with lead, suitable for cosmetic use.

### 2.4. FTIR Analysis

**Figure 2:**
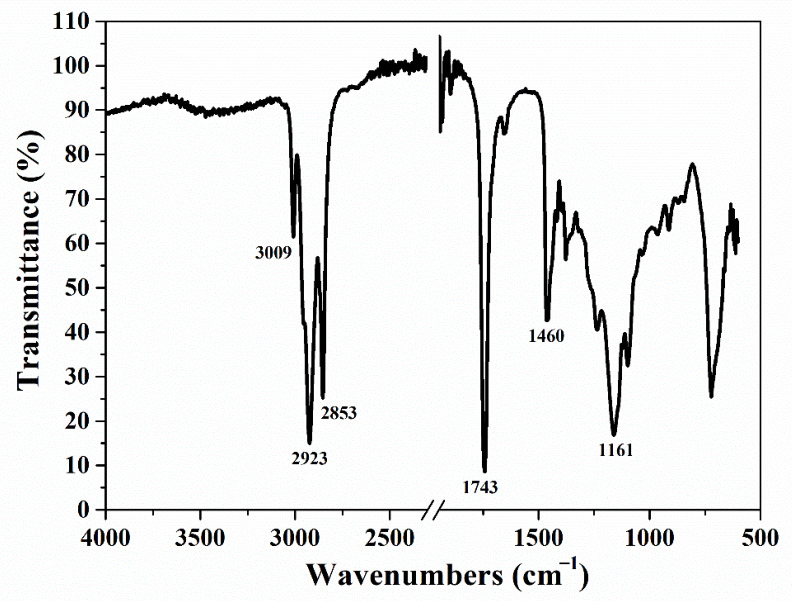
FTIR spectrum of the JRO sample.

The FTIR spectrum of JRO is represented in Figure 2.

The following main FTIR peaks are obtained for JRO: the triglyceride functional groups are found around 3009 cm^−1^ assigned to C–H bending scissoring, which represents the cis double-bond stretching; the peak at 2923 cm^−1^ is assigned to C–H asymmetric stretching vibration of CH_2_ (methylene group); the peak at 2852 cm^−1^ assigned to symmetrical stretching vibration of CH_2_ (methylene group); the peak at 1743 cm^−1^ which is assigned to C=O stretching from ester carbonyl functional group of the triglycerides; the peak at 1460 cm^−1^ is assigned to bending vibrations of the CH_2_ and CH_3_ aliphatic groups and the peak at 1161 cm^−1^ assigned to C-O stretching [45,46].

### 2.5. Analysis of Morphology by AFM Analysis

**Figure 3:**
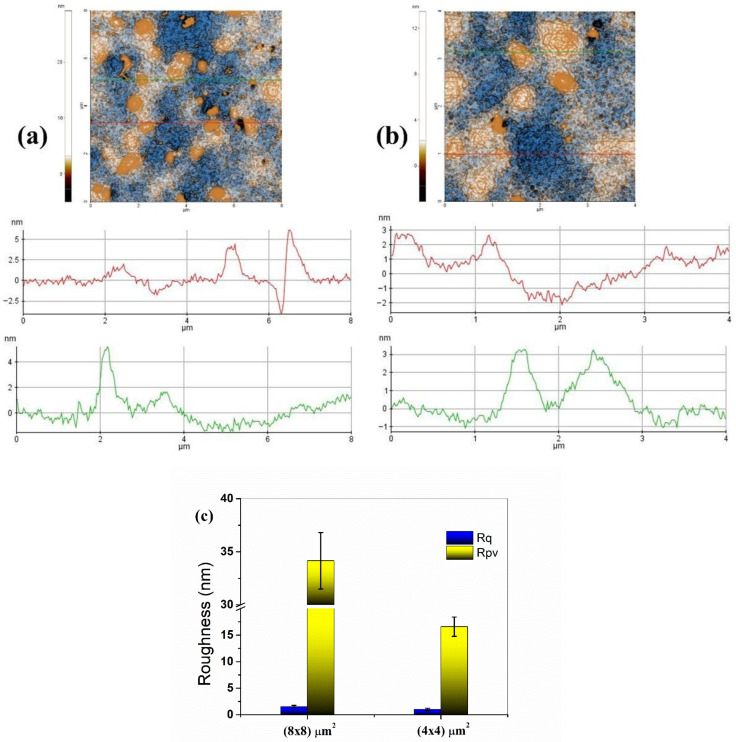
2D-AFM image and characteristic profiles (marked with red and green scan lines, below each AFM image) for JRO (**a**) The 2D-AFM images are scanned over areas of (8 × 8) µm^2^ and (**b**) (4 × 4) µm^2^. (**c**) Roughness (Rq) and peak-to-valley (Rpv) over the entire scanned areas: (8 × 8) µm^2^ and (4 × 4) µm^2^. Results are expressed with error bars drawn in the figures, calculated as standard errors (*n* = 3).

The 2D-AFM image of the JRO sample is displayed in Figure 3 (Figure 3a for scanned over an area of (8 × 8) µm^2^ and Figure 3b for scanned over an area of (4 × 4) µm^2^), and the characteristic line scan profiles are plotted at the position indicated by red and green lines.

JRO sample revealed a heterogeneous surface morphology, characterized by nanometric aggregates dispersed in a relatively uniform matrix. Height profiles indicated variations in the order of a few nanometers (3–5 nm), confirming the absence of a well-defined crystalline or fibrillar network. The RMS roughness (Rq) values of 1.531 nm for the whole area at a scale of (8 × 8) µm^2^ and 1.009 nm for the whole area at a scale of (4 × 4) µm^2^, respectively, suggest a globally smooth surface, while higher peak-to-valley parameter (Rpv) values of 34.157 nm for the whole area at a scale of (8 × 8) µm^2^ and 16.585 nm for the whole area at a scale of (4 × 4) µm^2^, respectively, indicate the presence of isolated prominent domains (Figure 3c). This structural behavior is consistent with GC–MS results, which revealed a high content of polyunsaturated fatty acids, especially linoleic acid, contributing to a fluid and poorly organized structural lipid system. The presence of phytosterols may favor local aggregation, without the formation of a continuous network.

### 2.6. Rheological Analysis

**Figure 4:**
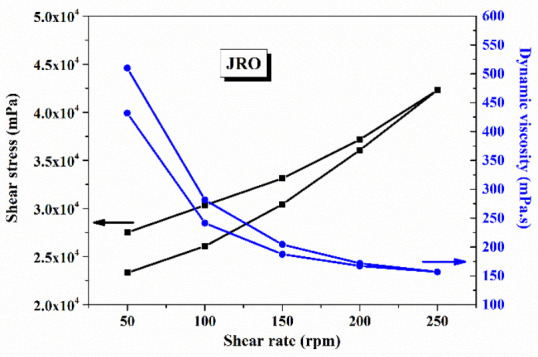
Shear stress (black line) and dynamic viscosity (blue line) variation function of shear rate in the JRO sample.

In Figure 4, the rheology measurements for JRO are shown.

**Figure 5:**
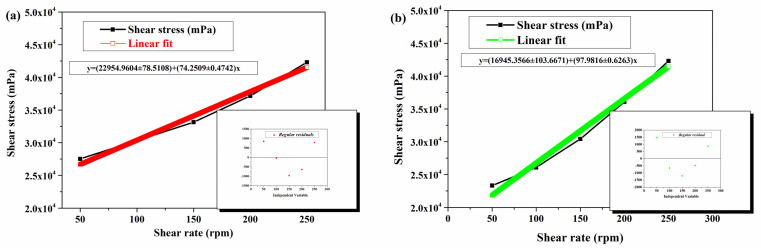
Linear fit of shear stress vs. shear rate for (**a**) up-shear rate and (**b**) down-shear rate (regular residual as insets).

According to Figure 4, it may be observed that the JRO dynamic viscosity depends on shear rates, especially for values below 150 rpm, and then slightly decreases with increasing shear rate, indicating a pseudoplastic behavior. In general, the edible vegetable oils exhibit Newtonian fluid behavior, characterized by a linear relationship between shear stress and shear rate, while dynamic viscosity remains constant over the entire shear range [47]. However, in our case of walnut oil with a higher content of unsaturated fatty acids (demonstrated by GS-MS analysis), deviations from ideal behavior occur, and it is manifested by a slight shear-thinning effect. Thus, the graphical representation of shear stress as a function of shear rate shows an approximately linear increase (Figure 5a,b, with regular residual as an inset), while the graph of dynamic viscosity as a function of shear rate indicates a slight decrease with increasing shear rate. These characteristics are essential for understanding the flow, processing, and application behavior of vegetable oils in various cosmetic or food formulations.

### 2.7. Spreadability Analysis

**Figure 6:**
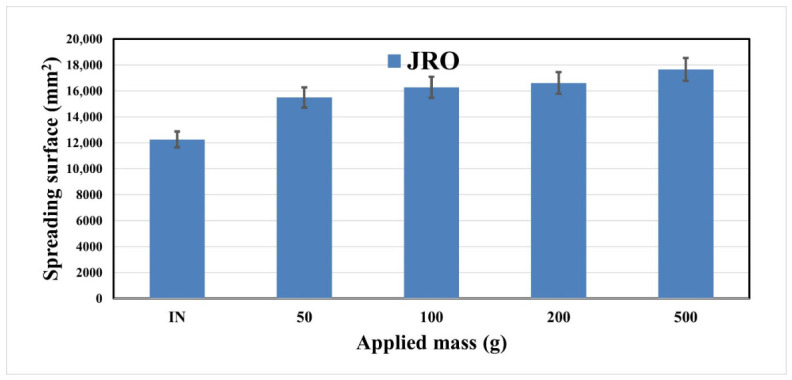
The spreading behavior of JRO. Results are expressed with error bars drawn in the figures, calculated as standard errors (*n* = 3).

Figure 6 shows the variation in the spreading surface with the applied mass for the JRO sample.

It was observed that the JRO sample has a higher spreadability, slightly increasing after the first 50 g of the applied mass. The result is correlated with the findings from AFM measurements, which demonstrate the smooth surface morphology. The higher content of polyunsaturated fatty acids, particularly linoleic acid (as demonstrated by the GS-MS analysis), contributes to a fluid–lipid matrix with reduced intermolecular interactions, facilitating easy spreading behavior [48].

### 2.8. Oxidation Stability

Lipid autoxidation is a major factor that can significantly reduce the shelf life and efficacy of cosmetic products, especially in the case of formulations rich in unsaturated oils or active ingredients sensitive to oxygen. The recorded induction period (IP) for JRO was 6.33 ± 0.05 h. The determined IP demonstrates a moderate oxidation resistance of JRO. The results are similar to other induction times determined in the literature for vegetable oils with a higher content of polyunsaturated fatty acids [49].

### 2.9. Antioxidant Activity

To evaluate the antioxidant activity of JRO, the samples were diluted in methanol in the following dilutions: 1:25, 1:10, 1:5, and 1:1, and compared with a solvent-based control and a JRO stock solution without dilution.

### 2.10. DPPH Method

**Figure 7:**
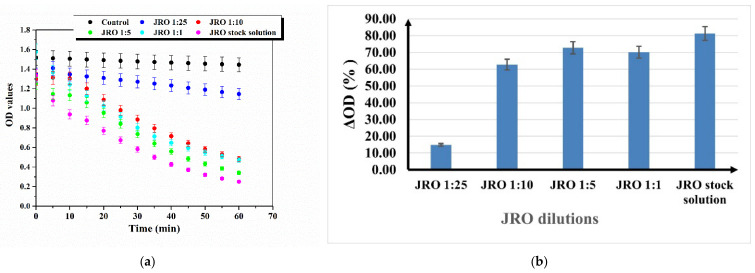
Antioxidant capacity determined from the DPPH assay for various dilutions of JRO. (**a**) Optical density (OD) values and (**b**) % decrease in optical density—ΔOD (%). Results are expressed with error bars drawn in the figures, calculated as standard errors (*n* = 3).

The level of antioxidant activity of the JRO was determined by spectrophotometric absorbance of DPPH reagent. The in vitro radical-scavenging capacity using the stable free radical 2,2-diphenyl-1-picrylhydrazyl (DPPH) of the JRO sample was represented in Figure 7a for optical density values versus various dilutions of JRO and Figure 7b as % decrease in optical density as a function of various JRO dilutions (4%, 10%, 20%, and 50% (*v*/*v*) of JRO in methanol, respectively).

From Figure 7a, it was observed that the highest OD values are obtained for the JRO 1:25 dilution, and the lowest value is obtained for the undiluted JRO sample. Regarding the antioxidant effect of JRO samples, an 81.3% was obtained for the undiluted sample, and then the antioxidant capacity diminishes with an increase in dilution. The JRO 1:1 diluted sample has a lower antioxidant capacity of 70.15% compared with JRO 1:5, which has the antioxidant capacity of 72.76%, and then the decreasing trend is maintained. In comparison with our previous study, where vitamin C was used as a positive control in the DPPH assay and exhibited 24.27% radical scavenging activity after 40 min, the JRO samples investigated in the present study showed higher DPPH radical scavenging activity (except for JRO 1:25 dilution), indicating a stronger antioxidant potential within this assay system [50].

### 2.11. ABTS Method

**Figure 8:**
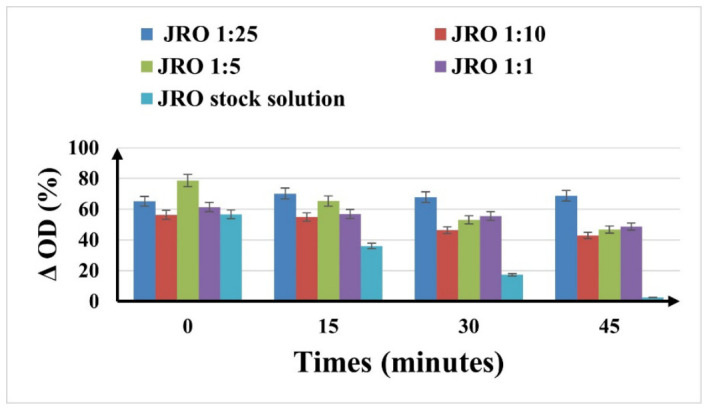
Antioxidant effect obtained using the ABTS method, determined as ΔOD (%) at different dilutions of JRO. Results are expressed with error bars drawn in the figures, calculated as standard errors (*n* = 3).

The evaluation of ABTS antioxidant capacity was performed at the same dilution as for the DPPH assay. The ABTS results are shown in Figure 8.

The antioxidant activity evaluated with the ABTS method showed a pronounced dependence on dilution and time. The antioxidant activity of the investigated JRO samples showed higher values compared to the positive control (vitamin C) previously reported in our studies [51], which exhibited an inhibition of 26.19% after 10 min. This suggests a strong radical scavenging capacity of the tested oil within the applied assay system. The JRO stock solution showed a rapid decrease in ΔOD (%) in time (from 56.63% at the initial time to 2.47% at 45 min), as an indication of intense neutralization of radicals. The maximum antioxidant activity was observed for JRO 1:5 dilution (78.61%), suggesting the existence of an optimal concentration for radical scavenging. For the JRO 1:10 dilution, a slight decrease in the antioxidant activity over time was observed, while for the JRO 1:25 dilution, the antioxidant effect remained more stable over time, probably due to slow kinetics in the consumption of active compounds. The observed results highlight a balanced effect between the antioxidant efficiency of JRO and its stability over time.

### 2.12. SPF Analysis

**Figure 9:**
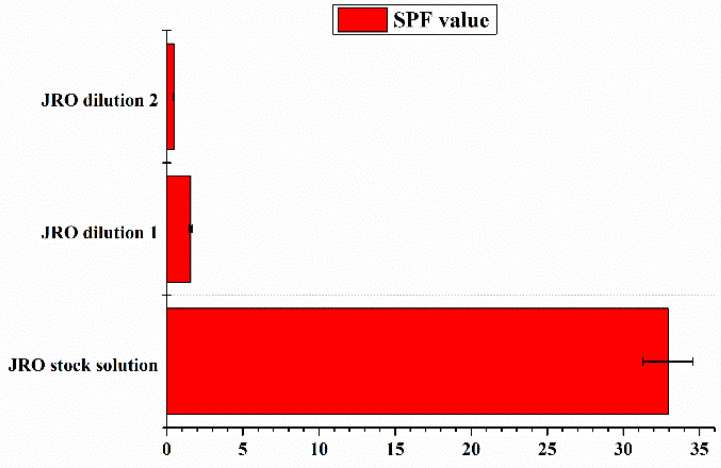
SPF determination of JRO stock solution and two diluted samples (JRO dilution 1 is a 1:100 dilution, and JRO dilution 2 is a 1:500 dilution). Results are expressed with error bars drawn in the figures, calculated as standard errors (*n* = 3).

The results from in vitro SPF analysis evaluated using the spectrophotometric method of Mansur, the JRO sample studied by the UV spectrophotometric method in the range of 290–320 nm, were presented in Table 6 and Figure 9.

It can be observed that the SPF value found for the JRO sample was 32.93 ± 1.65, and decreased with dilution. The SPF analysis demonstrates the efficiency of walnut oil to be used as a cosmetic raw material in sunscreen formulations to provide them with UV filters. Many liquid oils extracted from vegetable seeds and fruits (e.g., oils of avocado, olive, sesame, and almond) possess photoprotective activity against ultraviolet radiation (UV) [52].

Due to its high degree of unsaturation and susceptibility to oxidation, the integration of walnut oil into pharmaceutical formulations should be associated with the use of appropriate antioxidants in order to increase stability and maintain its functional properties.

## 3. Materials and Methods

### 3.1. Materials

**Figure 10:**
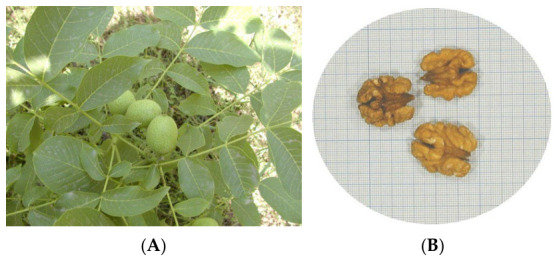
General aspect of (**A**) walnut fruits and (**B**) walnut seeds.

Walnut oil was obtained by cold pressing *Juglans regia* L. seeds (Figure 10A,B) harvested from Horezu, Romania (45°08′ N, 23°59′ E), between August and September 2024. The seeds were identified by the Department of Pharmaceutical Botany at the Faculty of Pharmacy, “Carol Davila” University of Medicine and Pharmacy, using standard methods. A voucher specimen is maintained in the Herbarium of the Botany Department, Faculty of Pharmacy, “Carol Davila” University of Medicine and Pharmacy (UBL 68/2024, Ph-UMFCD). Among methods for extracting vegetable lipids, cold pressing, although it yields a low amount, produces oils of superior quality that are slightly colored, have a pleasant odor, and a neutral reaction.

All chemicals used in experimental determinations were of analytical grade.

### 3.2. Synthesis of Walnut Oil

The synthesis method included the following steps:

- Drying the plant material (walnut kernels), with 100 g of vegetable product accurately weighed, to a moisture content of 4% using a fruit dryer (Zilan model ZLN-9645l, Istanbul, Turkey) at a residual pressure of 60–100 mm Hg and 40 °C;
- Grinding the plant material so that at least 60% of the particles pass through a 1 mm mesh stainless steel sieve (DIN ISO 3310-1) from NEXOPART GmbH & Co. K, Oelde, Germany, for homogenization;
- Pressing the plant material under increasing pressure in a continuously operating press (OlisLab 7100, produced by OLIS Ltd., Odessa, Ukraine);
- Purifying the crude oil, which was noted as JRO.

The purification step was a necessary process because the crude walnut oil obtained contains mechanical and organic impurities in suspension and traces of water that must be quickly removed to avoid degradation and to reduce losses. The following operations were performed to purify the pressed oil: (i) separation of coarse residues by centrifugation; (ii) elimination of traces of water by vacuum-drying to a moisture content of 0.05%; (iii) final filtration in a filter press. The extracted oil was kept in a cold medium in a dark bottle until its characterization process [53]. The selected temperature and pressure for the cold pressing process were (22 ± 2) °C and (760 ± 3) mm.

### 3.3. Methods for Characterization

The pH was measured using a calibrated ExStik pH meter (model PH110) (Extech, Taiwan, China), under controlled laboratory conditions. Density was determined by applying the classical formula, defined as the ratio of mass to volume, using precisely weighed and volumetrically measured oil samples. Each determination was made for three experiments, and the results are expressed as mean ± standard deviation.

The refractive index [54], relative density [55], acid value (mg KOH/1 g oil) [56], esterification (saponification) index (mg KOH/1 g oil) [57], and iodine index (I_2_ g/100 g oil) [58] of the oil sample were determined according to the European Pharmacopeia [27]. All analyses were performed in triplicate.

For GC-MS analysis, an Agilent Technologies 6890 N gas chromatograph equipped with an Agilent Technologies 5973 MSD mass detector (Agilent Technologies Inc., Santa Clara, CA, USA). The following method is useful for fatty acid determination: Lipids are saponified, and the resulting fatty acids are frequently transformed into methyl esters [59]. If the percentage of fatty acids is <5 g %, transmethylation (derivatization) is performed with sodium methoxide or NaOH (methanolic solution). Also, the separation and identification of compounds with hydroxyl and carboxyl groups (mono-, diglycerides, free fatty acids, phytosterols, alcohols, and phenols) in the form of volatile silylated derivatives by derivatization with bis-trimethylsilyltrifluoroacetamide was made. Silylation consists of introducing a trimethylsilyl group into a molecule by replacing an active hydrogen atom. This reduces the polarity of the compound, and thus, the silylated derivative is more volatile. Stability is also increased due to the reduction in the number of reactive sites containing active hydrogen. Silylated compounds are less polar, more thermally stable, and detection is better [60].

The reagents used are sodium hydroxide (60 g/L solution in methanol); anhydrous methanol, heptane, sodium chloride (200 g/L solution), anhydrous sodium sulfate; sodium chloride (saturated solution); bis-trimethylsilyltrifluoroacetamide (BSTFA); and heptane (Supelco/Sigma Aldrich Group, Darmstadt, Germany).

For fatty acid analysis, the following equipment was used: a water bath; a 25 mL round-bottom flask; a bubble condenser; Agilent Technologies 6890 N gas chromatograph (Agilent Technologies Inc., Santa Clara, CA, USA) with a DB-WAX polar capillary column (length 30 m, internal diameter 0.32 mm) with a stationary phase (polyethylene glycol, thickness 0.15 µm); and helium (as carrier gas) with a flow rate of 1.5 mL/min. For the analysis of silylated samples, the following was used: Agilent Technologies 6890 N gas chromatograph equipped with DB5-MS weakly polar capillary column (length 60 m, internal diameter 0.25 mm, stationary phase—phenylmethylpolysiloxane 5% with thickness 0.25 µm); helium (carrier gas) with a flow rate of 1.2 mL/min. For both analyses, an Agilent Technologies 5973 MSD mass detector (Agilent Technologies Inc., Santa Clara, CA, USA) with an electron impact ionization source (electron ionization energy—70 eV), a quadrupole mass analyzer, a splitless injector, and an autosampler was used.

Preparation of samples.

For the analysis of fatty acids, a quantity of 0.5 g of fatty oil is placed in a 25 mL round-bottom flask, and then 10 mL of anhydrous methanol and 0.2 mL of 60 g/L sodium hydroxide solution in methanol are added. The reflux condenser was attached and heated to boiling on a water bath. After 15 min, the flask was cooled under a water jet, and the contents were transferred to a separating funnel. The flask was rinsed with 4 mL heptane, transferred to the separating funnel, and 10 mL saturated sodium chloride solution was added under vigorous shaking. Then, the solution was left to stand for separation. The organic layer was transferred to a 10 mL volumetric flask by filtration over anhydrous sodium sulfate, then made up to the mark with heptane. A 1:5 dilution was made from the obtained solution. In total, 1 µL of the diluted sample was injected into the gas chromatograph.

For the analysis of silylated samples, 0.5 g of fatty oil was added to a derivatization vial, and then 0.5 mL of BSTFA derivatization reagent and 0.5 mL of heptane were added. The vial was sealed with a septum stopper and placed in a thermostated chamber, where it was heated for 1 h at 90 °C. After 2 h, 1 µL was injected into the gas chromatograph.

The samples were injected in triplicate for reproducibility.

The chromatographic procedure was as follows.

For fatty acid analysis, the solvent delay was 6.5 min, and the split ratio was 1:50; the injector temperature was set at 250 °C; the interface temperature was 280 °C; the ionization source temperature was 230 °C; the quadrupole temperature was 150 °C. The column temperature program was as follows: an initial temperature of 100 °C was held for 5 min, then the temperature was increased by two times with different values, first at a heating rate of 8 °C/min to 190 °C, and then at a rate of 3 °C/min to 220 °C, at which temperature it was maintained for 5 min.

For silylated sample analysis, the solvent delay was 6.5 min, and the split ratio was 1:10; the injector temperature was set at 250 °C; the interface temperature was—280 °C; ionization source temperature—230 °C; quadrupole temperature—150° C. The column temperature program was as follows: an initial temperature of 100 °C was held for 5 min, then the temperature was increased by two times with different values, first at 15 °C/min to 250 °C, and held at this temperature for 8 min, and then at 15 °C/min to 300 °C, which was maintained for 30 min.

The identification of the compounds was achieved by comparing the experimentally obtained mass spectra with those in the NIST database (https://www.agilent.com/en/product/software-informatics/mass-spectrometry-software/unknowns-analysis-by-gc-ms-method, accessed on 30 March 2026) and with those published in the specialized literature using ChemStation (1200 Series HPLC system) as a software program. The mass spectra were acquired in the range of 50–700 u.a.m. The chromatograms of the samples were quantitatively evaluated by the peak area normalization procedure. The percentage composition of the oils in fatty acids and other compounds was established by relating them to the total number of identified compounds.

### 3.4. Quantitative Determination of α-Linoleic Acids

The method consists of the transformation of α-linolenic acids in the form of methyl esters by refluxing the fatty oil with sodium hydroxide (NaOH) in methanol. Reagents: Sodium hydroxide (60 g/L solution in methanol); anhydrous methanol; heptane; sodium chloride (200 g/L solution); anhydrous sodium sulfate; sodium chloride (saturated solution); and standard substances (α-linolenic acid, 99%) are from Sigma, MP Biomedicals.

Preparation of the sample: Masses between 90 and 900 mg of fatty oil were analyzed and processed according to the above preparation of samples for fatty acids method. A 1:5 dilution is made from the obtained solution. Then, 1 µL of the sample was injected into the gas chromatograph.

Preparation of reference: 50 mg of α-linolenic acid was weighed and then processed according to the above preparation of samples for fatty acids method, respectively. The separated organic layer is collected in a 5 mL volumetric flask and made up to the mark with heptane (stock solutions with a concentration of 10 mg/mL are obtained).

Calibration curves: From each stock solution, five calibration solutions of α-linolenic acid in a mixture are prepared by dilutions corresponding to the concentration range of 20–1000 µg/mL. 1 µL of each calibration solution was injected. The obtained data were used to draw the calibration curves (linear regression lines) by plotting the dependence of the chromatographic response (peak area) on the concentration.

Working method:

In total, 1 µL of each diluted sample solution was injected, and the chromatogram was recorded (*C*_1_ µg/mL). The percentage concentration (mg α-linolenic acid/100 g fatty oil) is calculated with the following formula:

(1) $$C=\frac{C_{1}}{m\;\times 1000}\times 50\times 100$$

where *C*_1_—concentration of α-linolenic acid resulting from the equation of the calibration curve (expressed in µg/mL), *m* is the amount of fatty oil analyzed (mg), and 50 is the dilution factor.

### 3.5. Heavy Metal Content by GFAAS Analysis

Heavy metals, arsenic (As) and lead (Pb), were identified using graphite furnace atomic absorption spectrophotometry (GFAAS) [53]. The mineralization of the walnut oil sample was made using the Ethos Easy microwave digestion system (Milestone SpA, Sorisole (BG), Italy). Approximately 0.300 g of sample, 1 mL of H_2_O_2_ (30%), 9 mL of HNO_3_ (69%), and HPLC-grade water were brought into a vial to obtain the detection of impurities at the ppb (µg/L) level. The oven is equipped with five mineralization segments/vials, corresponding to the positions 1, 6, 7, 8, and 11. The sample is weighed on the analytical balance directly in the vial, after which 1 mL of 30% hydrogen peroxide and 9 mL of 69% nitric acid are added, according to the mineralization method selected from the existing library in the equipment operating (easyCONTROL v.3), namely the Palm Oil software. It is recommended that segment 1, where the sensor (thermocouple for temperature monitoring and control up to 300° C) is inserted, contain one of the samples of interest. If an exothermic chemical reaction occurs, the sample will be placed in segment 1.

The first stage of the mineralization process lasts 15 min and involves increasing the temperature up to 200° C, for five segments, with the maximum power used being 1800 W.

The second stage, also lasting 15 min, consists of maintaining the temperature at 200 °C. at the same power. After mineralization is complete, the vials are cooled using the apparatus fans for approximately 10–15 min. Subsequently, the segments can be removed from the device. The mineralized samples can then be analyzed by atomic absorption spectrometry, using the graphite furnace method. Elemental analysis was performed by graphite furnace atomic absorption spectrometry (GFAAS), using linear calibration curves and constant blank verification. The results indicate a low lead level, characteristic of high-purity vegetable oils.

All solutions were prepared with high-purity deionized water obtained with a Millipore system. Glassware and polyethylene bottles were cleaned by immersion in 10% nitric acid (Merck, Suprapur, Darmstadt, Germany), followed by three rinses with deionized water. High-purity concentrated nitric acid (69%, Trace Grade analysis) was purchased from Merck.

### 3.6. Preparation of As and Pb Calibration Solutions

A high-purity arsenic stock standard solution with a concentration of 1000 mg/L H_3_AsO_4_ in 0.5 M nitric acid was purchased from Merck. A volume of 10 mL was taken from the stock solution, and successive dilutions were made to obtain a final concentration of 100 µg/mL, which was used to prepare the 2, 4, 6, 8 and 10 µg/L standards.

Similarly, the high-purity lead stock standard solution with a concentration of 1000 mg/L Pb(NO_3_)_2_ in 0.5 M nitric acid was purchased from Merck. A volume of 10 mL of the initial stock solution was successively diluted to a final concentration of 100 µg/mL and used to prepare the standards of 2, 4, 6, 8, and 10 µg/L.

The determinations were made using a Thermo Electron Inc. SOLAAR 6M atomic absorption spectrometer (Thermo Electron Inc., Waltham, MA, USA) equipped with a deuterium lamp for background correction. The analysis using the GFAAS technique (using the GF95Z Zeeman graphite furnace system and FS95 autosampler) from Thermo Electron Inc. was performed. The working wavelength for As was 193.7 nm, and for Pb was 283.3 nm, respectively. For the GFAAS technique, a graphite pyrolytic cuvette was used; the sample volume and the volumes of the injected standards were 20 µL. The height of the autosampler capillary tip in the cuvette was adjusted by observing the injection using a camera positioned in the graphite furnace (GFTV), the accessory being provided with the spectrometer.

All measurements were performed with at least two replicate determinations for each sample, using integrated absorbance.

Argon was used as a shielding gas throughout the determination, with a flow rate of 0.2 L/min. The performance of our GFAAS method for metal determination was demonstrated using standard samples.

The 4 stages in the temperature program are: (i) stage 1—drying; (ii) stage 2—pyrolysis; (iii) stage 3—atomization and determination, and (iv) stage 4—cleaning. The As and Pb concentrations in each oil sample were calculated from the corresponding regression line, namely, absorbance as a function of concentration.

To study the applicability range of the GFAAS method, the calibration curves using As and Pb standards for the following concentration ranges: 2, 4, 6, 8, and 10 µg/L were plotted. The linear equations of the calibration curves have the following formula: for As: y = 0.0189x + 0.0041, with a correlation coefficient r^2^ = 0.9992; for Pb: y = 0.0306x + 0.0033, with a correlation coefficient r^2^ = 0.9991.

### 3.7. FTIR Analysis

Infrared spectroscopic measurements were performed using the NICOLET 6700 FT-IR spectrophotometer (Thermo Electron Corporation, Waltham, MA, USA) with Fourier transform (FT-IR), in transmission in the range of 4000–500 cm^−1^. The oil sample was applied as a drop onto KBr windows, forming a thin film suitable for IR transmission analysis, at room temperature. The spectra were recorded at a resolution of 4 cm^−1^ and processed using the OMNIC 7.3 software.

### 3.8. AFM Analysis

Oil samples (20 µL each) were diluted in 2 mL 96% ethanol (Merck Millipore, Burlington, MA, USA) and deposited onto clean glass substrates. Then, they were heated at 30 °C for 30 min to ensure good adhesion and solvent evaporation. The AFM images were acquired in contrast-enhanced mode, and the surface profiles, highlighted by line scans displayed below the images, clearly illustrate the topography of both oil samples, according to the method previously described in our study [61].

### 3.9. Spreadability Study

The spreading behavior of the analyzed oil sample was evaluated using an extensometer (Epsilon Technology Corp., Jackson, WY, USA). Two glass plates were used for the determinations; a quantity of 0.5 g of oil was placed in the center of the lower plate. Subsequently, the upper plate, with a weight of 150 g, was added, and the diameter of the oil stretching zone was measured. Next, additional weights of 50, 100, 200 and 500 g were gradually applied, and after a resting time of 1 min for each step, the diameter of the surface occupied by the oil sample was determined [62].

The spreading area was calculated using Equation (2):

(2) $$S\;=\;\pi r^{2}$$

where *S* is the spreading area (mm^2^), and *r* is the radius (mm).

### 3.10. Oxidative Stability Test

To evaluate the oxidative stability, expressed by the induction period, of the studied oil sample, the Velp OXITEST reactor (Velp Scientifica, Usmate, Italy) was used, which accelerates the oxidation process under controlled and reproducible conditions. The test consists of exposing oil samples to high oxidative stress by subjecting them to a temperature of 90 °C and a pressure of 6 atm, in the presence of pure oxygen. Small quantities of product (10 g) were introduced into the oxidation chamber, where the oxygen consumption was continuously monitored. As the oxidation reaction progresses, oxygen is consumed, causing the internal pressure to decrease. The moment at which a sudden drop in oxygen pressure is observed, indicating the onset of significant oxidation, was determined using the OXISoft™ 3.0.0 software, integrated into the Velp OXITEST system. The induction period (IP) is defined as the time interval (expressed in hours and minutes) until this sharp drop in oxygen pressure occurs, signaling the initiation of advanced oxidation. This point is correlated with the formation of primary oxidation products and is associated with the appearance of the first signs of rancidity or changes in sensory and functional properties.

Two calculation methods were used to determine the IP:

(i) The least squares method, which involves fitting a curve based on oxygen consumption data.

(ii) The graphical method was used as an additional recalculation approach when it was necessary. A longer induction period indicates an increased resistance of the oil to oxidative degradation and, implicitly, a better stability under real storage conditions. This parameter is particularly important for formulations containing vegetable oils, unsaturated fatty acids, or antioxidants, and is essential in establishing the optimal packaging, storage, and formulation optimization conditions.

### 3.11. Rheological Analysis

The rheological experiments were carried out on a B One Plus rotary viscometer (Lamy Rheology, Champagne-au-Mont-d’Or, France), with an accuracy of ±1% of full scale and a repeatability of 2%. The apparatus is equipped with seven probes (spindles), labeled RV1-RV7, each covering a specific viscosity range. The RV3 probe was used to determine oil viscosity by immersing it in 50 mL of each sample. The measurements were performed at various rotation speeds of 50, 100, 150, 200, and 250 rpm for 10 s for each rotation speed while the temperature remained constant (at 22 °C).

### 3.12. Antioxidant Activity

DPPH method.

The antioxidant activity of JRO was tested in terms of radical-scavenging capacity using the stable free radical 2,2-diphenyl-1-picrylhydrazyl (DPPH) (Sigma-Aldrich, St. Louis, MO, USA), as previously described [63,64]. Solution of DPPH in methanol was added to the tested JRO sample at several dilutions in methanol (1:1, 1:5, 1:10, and 1:25). The corresponding concentrations were 4%, 10%, 20%, and 50% (*v*/*v*) of JRO in methanol. The 1:2 ratio was kept between the JRO sample and the DPPH solution. The absorbance values were measured at 517 nm over 1 h. Several incubation periods of 5, 30, and 60 min were analyzed to determine the AA%, named as Δ*OD* (%) (Equation (3)):

(3) $$∆OD\left(\%\right)=\left(\frac{{OD}_{sample}-{OD}_{stock\;solution}}{{OD}_{control}}\right)\times 100$$

where *OD_sample_* was the *OD* of the sample solution with DPPH, *OD_stock_*_solution_ was the absorbance of the sample solution without the DPPH, and *OD_control_* was the solvent-based standard (the absorbance of the DPPH solution). All analyses were undertaken on three replicates, and the results were averaged.

ABTS method.

The ABTS (2,2′-Azino-bis(3-ethylbenzothiazoline-6-sulfonic acid)) method is based on measuring the sample’s scavenging capacity of the ABTS+∙free radical. The stock reagent containing ABTS 7 mM and K_2_S_2_O_8_ 2.45 mM was kept in the dark for 16 h at 4 °C for the generation of the green ABTS+∙ free radical. The solutions used for this method were obtained by diluting the stock solution with distilled water until an appropriate OD (0.8) was reached. The wavenumber was set at 714 nm. The incubation period was 10 min.

Samples noted as 1:1, 1:5, 1:10, and 1:25 dilutions were incubated with ABTS WS in a 1:3 ratio. A control was prepared using methanol. Results are presented as the % decrease in optical density (Δ*OD*) as follows:

(4) $$∆OD\left(\%\right)=\left(\frac{{OD}_{control}-{OD}_{sample}}{{OD}_{control}}\right)\times 100$$

and directly correlated to the antioxidant capacity of the JRO [65].

### 3.13. Sunscreen Properties

In total, 1 mL of each JRO was added to a 100 mL volumetric flask, diluted with ethanol to the mark, and ultrasonicated for 5 min. The in vitro determination of the sun protection factor (SPF) was made using a Perkin-Elmer Lambda 35 UV-Vis spectrophotometer (Perkin-Elmer Inc., Waltham, MA, USA). Oil samples were placed in microcuvettes with a 10 mm light path, and absorbance spectra were recorded over 290–320 nm at every 5 nm intervals. SPF can be evaluated by applying the Mansur equation [66,67,68]:

(5) $$SPF=CF\times \sum_{290}^{320}EE\left(\lambda \right)\times I\left(\lambda \right)\times Abs\left(\lambda \right)$$

where *CF*—correction factor (10), *EE*(*λ*)—erythemogenic effect of radiation with wavelength *λ*, *I*(*λ*)—the intensity of solar light at wavelength *λ*, and *Abs*(*λ*)—the absorbance of wavelength *λ*. The method is briefly reported in our previous study [53].

### 3.14. Statistical Analysis

All measurements were performed in triplicate to ensure reproducibility, and the results are expressed as mean ± standard deviation (SD). Error bars are drawn in the figures, being calculated as standard errors or standard deviations.

## 4. Conclusions

This study elucidates the chemical composition, morphological, spectral, physicochemical, rheological properties, and antioxidant activity of JRO extracted from walnut seeds, highlighting its potential as a cosmetic raw material. GC–MS analysis showed the predominance of polyunsaturated fatty acids, especially linoleic acid, followed by α-linolenic acid. AFM analysis revealed a relatively smooth surface, with a heterogeneous organization, aspects specific to a fluid lipid system rich in unsaturated fatty acids. Antioxidant activity determined by the DPPH and ABTS methods demonstrated a dependence on concentration and time for the JRO sample. The maximum effect was observed at a dilution of 1:5, indicating an optimal concentration for free radical scavenging. At high concentrations (low dilutions), antioxidant activity decreased rapidly over time, suggesting a rapid reduction of active compounds. In contrast, at low concentration (high dilutions), activity was more stable, indicating slower reaction kinetics and gradual consumption of antioxidants. Overall, the results highlight an equilibrium between antioxidant efficiency and stability, influenced by the chemical composition and structural organization of the lipid system. In conclusion, the cold-pressed walnut oil (JRO) presents adequate physicochemical and structural properties for cosmetic integration. Although the arsenic content slightly exceeds the threshold for food consumption, the levels are compliant with safety requirements for topical cosmetic products. This supports its use as a sustainable, natural ingredient in sunscreen and skincare formulations without compromising consumer safety. However, due to its high content of polyunsaturated fatty acids and susceptibility to oxidative and photooxidative degradation, it is recommended to improve stability by adding additional photostabilizers and antioxidants with complementary action in the final formulations.

## Figures and Tables

**Table 1:** Physical and chemical properties of JRO.

Parameters	Values *
Refractive index	1.4719 ± 0.0200
Density (at 25 °C) (g/cm^3^)	0.929 ± 0.030
pH	5.00 ± 0.02
Viscosity (^o^Engler)	9.22 ± 0.01
Acidity index (mg KOH/1 g oil)	7.00 ± 0.03
Saponification index (mg KOH/1 g oil)	133.90 ± 0.05
Iodine index (I_2_ g/100 g oil)	76.90 ± 0.02

* Results are expressed as mean ± SD (*n* = 3).

**Table 2:** Results of GC-MS analysis of fatty acids from JRO.

Fatty Acid		Retention Time (min)	Area (%)
Palmitic Acid	C16:0	14.558	6.394
Stearic acid	C18:0	16.870	1.905
Oleic acid	C18:1	17.046	20.019
10-Octadecenoic acid	C18:1	17.126	0.847
Linoleic acid	C18:2	17.580	58.582
α-Linolenic acid	C18:3	18.362	12.253
Total identified fatty acids			100%

**Table 3:** GC-MS analysis results of the silylated oil JRO sample.

Compound	Retention Time (min)	Area (%)
1-dodecyne	11.863	0.060
2,4-decadienal	12.147	0.210
free palmitic acid	18.804	6.832
octadecatriene	19.231	0.616
docosatriene	19.286	0.413
free linoleic acid	21.205	41.679
free oleic acid	21.284	22.630
free linoleic acid	21.324	3.210
free 11-octadecenoic acid	21.387	2.886
free stearic acid	21.679	2.588
free 11-eicosenoic acid	24.901	1.721
free arachidonic acid	25.240	0.316
free 8,11,14-eicosatrienoic acid	25.777	1.427
free trichosenic acid	27.507	2.811
free 2-methyl-2-p-methoxymandelic acid	33.287	2.913
β-sitosterol	42.242	8.588
stigmasterol	42.732	1.101
Total identified compounds		100%

**Table 4:** Results of α-linolenic acid dosage.

Compound	Mass of Fatty Acid (mg)	α-Linolenic Acid		
		Retention Time (min)	c_1_ (µg/mL)	C (mg/100 g)
JRO	502.82	19.12	251.47	2.50

c_1_—concentration of α-linolenic acid resulting from the equation of the calibration curve (expressed in µg/mL); C is the percentage concentration (mg α-linolenic acid/100 g fatty oil).

**Table 5:** Metal concentrations in the JRO sample.

Metal	Oil Sample	Sample Weight (g)	Metal Concentration	
			µg/L	µg/g
As	JRO	0.342	4.968	0.145
Pb		0.342	1.624	0.047

**Table 6:** Absorbance of JRO samples.

Wavelength (nm)	EE (λ) × I (λ)	JRO Stock Solution	JRO Dilution 1 *	JRO Dilution 2 **
290	0.015	3.339 ± 0.098	0.278 ± 0.007	0.035 ± 0.002
295	0.0817	3.388 ± 0.095	0.223 ± 0.010	0.034 ± 0.001
300	0.2874	3.345 ± 0.085	0.197 ± 0.011	0.039 ± 0.004
305	0.3278	3.261 ± 0.120	0.148 ± 0.014	0.047 ± 0.005
310	0.1864	3.233 ± 0.132	0.106 ± 0.015	0.055 ± 0.008
315	0.0837	3.289 ± 0.099	0.098 ± 0.013	0.055 ± 0.005
320	0.018	3.231 ± 0.077	0.066 ± 0.010	0.054 ± 0.006

* JRO Dilution 1 is 1:100 dilution, and ** JRO Dilution 2 is 1:500 dilution. Data are expressed as mean ± SD (*n* = 3).

## Data Availability

The original contributions presented in this study are included in the article. Further inquiries can be directed to the corresponding author.
